# Case Volume Benchmarks During Residency and Fellowship Training for Pediatric Orthopedic Surgeons

**DOI:** 10.7759/cureus.32738

**Published:** 2022-12-20

**Authors:** Jason Silvestre, John M Flynn, Terry L Thompson, Matthew E Oetgen

**Affiliations:** 1 Orthopaedic Surgery, Children's National Hospital, Washington, USA; 2 Orthopaedic Surgery, Children's Hospital of Philadelphia, Philadelphia, USA; 3 Orthopaedic Surgery, Howard University College of Medicine, Washington, USA

**Keywords:** accreditation council for graduate medical education (acgme), fellowship training, orthopedics surgery, paediatric orthopedics, pediatric fractures

## Abstract

Higher case volumes correlate with improved clinical outcomes across surgical specialties. This study establishes case volume benchmarks after completion of pediatric orthopedic fellowship training. Case logs for orthopedic surgery residents and pediatric orthopedic fellows at accredited programs were analyzed (2017-2018 to 2020-2021). Case volumes for pediatric orthopedic surgery were compared using parametric tests. Case logs from 3,000 orthopedic surgery residents and 149 pediatric orthopedic fellows were analyzed. There was an increase in total pediatric cases among orthopedic surgery residents over the study period (273 ± 68 to 285 ± 76, 1.1% annual increase, P<0.001). On average, pediatric orthopedic fellows reported 276 cases: Spine deformity (55 cases, 20%), foot and ankle deformity (45 cases, 16%), hip reconstruction (34 cases, 12%), limb deformity (32 cases, 12%), trauma lower limb (24 cases, 9%), treatment of supracondylar humerus fracture (23 cases, 8%), trauma upper limb (19 cases, 7%), clubfoot (18 cases, 7%), soft tissue procedures (13 cases, 5%), open treatment of femoral shaft fractures (6 cases, 2%), and treatment of infection (7 cases, 3%). Pediatric orthopedic fellows effectively doubled their pediatric case volume from fellowship training. The distribution of pediatric orthopedic fellow case volume percentiles was: 10^th^ - 191 cases; 30^th^ - 237 cases; 50^th^ - 275 cases; 70^th^ - 318 cases; 90^th^ - 382 cases. Case volume benchmarks can help inform orthopedic trainees, faculty, and patients about the impact of pediatric orthopedic fellowship training. More research is needed to elucidate optimal training environments for future pediatric orthopedic surgeons.

## Introduction

Orthopedic surgery residents must develop and demonstrate clinical competency across both adult and pediatric management of the musculoskeletal disease. The Accreditation Council for Graduate Medical Education (ACGME) outlines Milestones for orthopedic surgery resident competencies across total joint reconstruction, foot and ankle, hand surgery, musculoskeletal oncology, sports medicine, trauma, spine surgery, and pediatric orthopedics [1]. Given the unique skills necessary for pediatric care, virtually all orthopedic surgeons who want pediatrics to be a significant portion of their future practice pursue pediatric orthopedic fellowship training [2,3].

Currently, the ACGME requires orthopedic surgery residents to perform 1,000 total cases to graduate from residency, of which 200 are required in pediatric orthopedics [4]. Reported case volumes are closely monitored during residency and summarized in ACGME case logs for future employment and accreditation purposes. As of the 2021-2022 academic year, ACGME-accredited pediatric orthopedic fellowships did not have case minimum requirements for graduation.

Yet, at the same time, there is a clear association between increased operative experience and improved clinical outcomes across surgical disciplines [5,6], including those involving children [7-15]. Increasingly, hospital administrators have made attending privileges contingent on fellowship training and case minimums [5-6]. To date, few studies have assessed reported case volumes during pediatric orthopedic fellowship training. A review of ACGME case logs for pediatric orthopedic fellows in the years 2012 to 2015 demonstrated marked variability in reported case volume with over a three-fold difference between the 10^th^ and 90^th^ percentiles of fellows by case volume [16]. Understanding contemporary trends in reported case volumes during pediatric orthopedic fellowship training may assist orthopedic surgery residents to plan for a career in pediatric orthopedic surgery. National governing bodies in orthopedic surgery education can also use these data to identify potential deficiencies in pediatric orthopedics training.

This study elucidates case volume benchmarks for ACGME-accredited pediatric orthopedic fellowship training. We elucidate the impact of pediatric orthopedic fellowship training on reported pediatric cases relative to the volume of pediatric cases performed during orthopedic surgery residency. Ultimately, we define case volume benchmarks at the conclusion of surgical training to inform orthopedic surgery trainees, faculty, and patients on the impact of pediatric orthopedic fellowship training.

## Materials and methods

The ACGME provided case logs for pediatric orthopedic fellows and orthopedic surgery residents who graduated during the 2017-2018 to 2020-2021 academic years. ACGME-accredited pediatric orthopedic fellowship training programs were standardized at one year of duration, performing exclusively pediatric orthopedic surgeries. ACGME case logs summarize cumulative operative experience at the end of a training period and are an amalgamation of primary assigned credits to a particular case. Thus, in cases where multiple procedural codes were assigned to a case, only the trainee-marked primary credit was assigned. Cases are self-reported and closely monitored by program directors and faculty on a semi-annual basis. Periodically, case logs are also audited by the ACGME during the accreditation process. This study qualified for review exemption based on the policies of the Institutional Review Board at Howard University.

ACGME case logs summarize reported case volumes for accredited pediatric orthopedic fellowship programs. Thus, non-accredited fellowships were excluded from this study. Within pediatric orthopedic fellowship training, the majority of programs are ACGME accredited [2].

Reported case categories for pediatric orthopedic fellowships are a collection of Current Procedural Terminology (CPT) codes that are defined by the ACGME [17]. From the 2017-2018 academic year to present, pediatric orthopedic surgery case categories were refined from generic case categories used for orthopedic surgery residency to specific case categories for pediatric orthopedic fellowship training. As such, study inclusion was limited from 2017-2018 to 2020-2021 to facilitate meaningful multi-year comparisons. Total pediatric cases performed during orthopedic surgery residency were compared with total cases performed during pediatric orthopedic fellowship.

Descriptive statistics were calculated on GraphPad Prism 6 (San Diego, CA). Annual growth rates in pediatric cases during orthopedic surgery residency were calculated to elucidate trends in reported case volumes over the study period. Operative benchmarks for pediatric orthopedics fellowship training were calculated via weighted averages for fellow percentiles of reported case volumes over the study period. Parametric tests were used to compare reported case volumes during orthopedic surgery residency and pediatric orthopedic fellowship. Reported case volumes were summarized by case category as means ± standard deviations (SDs). P values of < 0.05 were significant.

## Results

ACGME-accredited pediatric orthopedic surgery fellowship training

During the 2021-2022 academic year, there were 46 pediatric orthopedic fellowships advertised on the POSNA Fellowship Directory, of which 24 were ACGME accredited (52%). One hundred forty-nine ACGME-accredited pediatric orthopedic fellows and 3,000 orthopedic surgery residents were included in this study (Table 1). The annual number of orthopedic surgery residents increased from 729 in 2017-2018 to 822 in 2020-2021 (a 3% annual increase). The annual number of pediatric orthopedic fellows decreased from 40 in 2017-2018 to 34 in 2020-2021 (a -4% annual decrease).

**Table 1:** Number of Residents and Fellows in ACGME Accredited Pediatric Orthopedic Fellowship and Orthopedic Surgery Residency Training

Academic Year	Orthopedic Surgery Residency		Pediatric Orthopedic Fellowship	
	Number of Programs	Number of Residents	Number of Programs	Number of Fellows
2017-2018	154	729	21	40
2018-2019	154	725	19	37
2019-2020	154	724	23	38
2020-2021	180	822	19	34
Total	--	3,000	--	149

Trends in Pediatric Cases Reported During Orthopedic Surgery Residency

**Figure 1:**
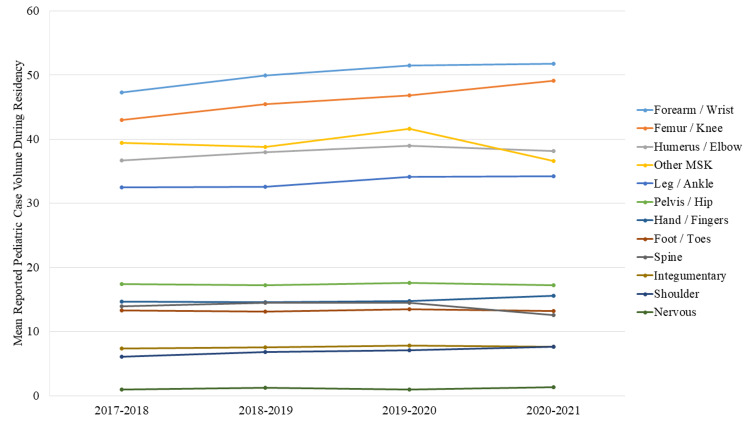
Total Pediatric Case Volume Reported During Orthopedic Surgery Residency *MSK: musculoskeletal

The total reported pediatric orthopedic surgery case volume during residency increased from 273 ± 68 to 285 ± 76 over the study period ([Table 2], 1% annual increase, P<0.001). This was driven primarily by more cases in the forearm/wrist and femur/knee (Figure 1). Pediatric case categories that experienced the highest growth rates in reported case volumes were nervous system (1.0 ± 2 to 1.3 ± 2, 7% annual increase, P=0.005), shoulder (6 ± 4 to 8 ± 6, 6% annual increase, P<0.001), and femur/knee (43 ± 17 to 49 ± 23, 3% annual increase, P<0.001).

**Table 2:** Pediatric Cases Reported During Orthopedic Surgery Residency Training * ANOVA test was used to compare annual reported case volumes

Case Categories	Average Number of Reported Cases ± SD				Annual growth rate, %	P
	2017-2018	2018-2019	2019-2020	2020-2021		
Shoulder	6 ± 4	7 ± 5	7 ± 5	8 ± 6	6%	<0.001
Humerus/Elbow	37 ± 15	38 ± 16	39 ± 18	38 ± 16	1%	0.058
Forearm/Wrist	47 ± 23	50 ± 25	52 ± 27	52 ± 22	2%	0.001
Hand/Fingers	15 ± 9	15 ± 10	15 ± 10	16 ± 10	2%	0.157
Pelvis/Hip	17 ± 9	17 ± 10	18 ± 10	17 ± 10	-0.3%	0.836
Femur/Knee	43 ± 17	46 ± 20	47 ± 21	49 ± 23	3%	<0.001
Foot/Toes	33 ± 13	33 ± 13	34 ± 15	34 ± 13	1%	0.014
Leg/Ankle	13 ± 8	13 ± 8	14 ± 8	13 ± 8	-0.2%	0.802
Other Musculoskeletal	39 ± 32	39 ± 31	42 ± 32	37 ± 30	-2%	0.019
Spine	14 ± 13	15 ± 12	15 ± 15	13 ± 13	-2%	0.013
Integumentary System	7 ± 6	8 ± 7	8 ± 8	8 ± 7	1%	0.733
Nervous System	1 ± 2	1 ± 2	1 ± 2	1.3 ± 2	7%	0.005
Total	273 ± 68	280 ± 73	289 ± 84	285 ± 76	1.1%	<0.001

Distribution of cases reported during pediatric orthopedic fellowship training

**Figure 2:**
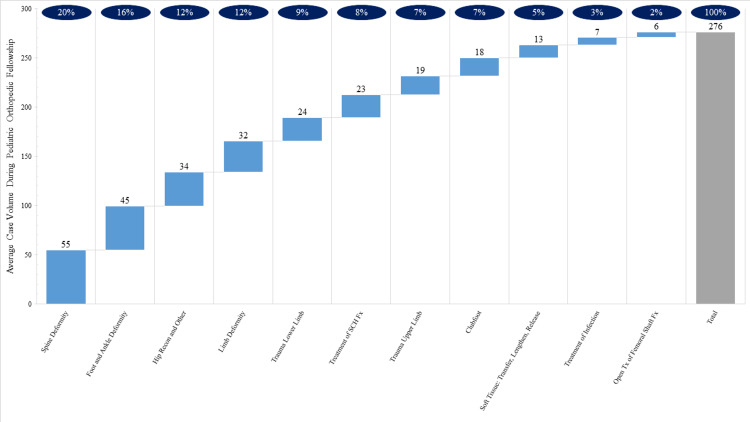
Distribution of Case Volume Reported During ACGME Accredited Pediatric Orthopedic Fellowship Training *Weighted average of case volumes reported between 2017-2018 to 2020-2021

Figure 2 is a waterfall chart showing the distribution of reported cases during pediatric orthopedic fellowship training over the study period. Pediatric orthopedic fellows reported an annual average of 276 ± 78 total cases, of which the majority were spine deformity (20%), foot and ankle deformity (16%), and hip reconstruction and other (12%). A minority of cases were trauma of the lower limb (9%) and treatment of supracondylar humerus fracture (8%). Only 2% of cases were open treatment of femoral shaft fracture.

Comparison of cases reported during residency and pediatric orthopedic fellowship training

On average, orthopedic surgery residents performed 282 ± 80 pediatric cases at the conclusion of residency. Pediatric orthopedic fellows reported an average of 276 ± 78 total cases. Thus, pediatric orthopedic fellows effectively doubled their case volume in pediatric orthopedic surgery with one year of fellowship training.

Operative benchmarks during pediatric orthopedic fellowship training

Percentiles were calculated for reported case volume during pediatric orthopedic fellowship training (Table 3). Over the study period, the distribution of fellow percentiles for total pediatric case volumes were as follows: 10^th^ - 191 cases; 30^th^ - 237 cases; 50^th^ - 275 cases; 70^th^ - 318 cases; 90^th^ - 382 cases.

**Table 3:** Case Volume Benchmarks for Pediatric Orthopedic Fellowship Training *Percentiles based on weighted averages over the study period (2017-2018 to 2020-2021); foot and ankle deformity excludes clubfoot

Pediatric Case Category	Fellow Percentiles for Reported Case Volumes				
	10^th^	30^th^	50^th^	70^th^	90^th^
Spine Deformity	12	28	45	64	106
Foot and Ankle Deformity	16	28	36	51	84
Hip Reconstruction and Other	17	25	33	42	56
Limb Deformity	13	23	28	37	57
Trauma Lower Limb	11	17	23	29	39
Treatment of Supracondylar Humerus Fracture	9	15	21	29	41
Trauma Upper Limb	9	14	18	25	32
Clubfoot	1	6	11	22	44
Soft Tissue: Transfer, Lengthen, Release	2	4	7	16	31
Treatment of Infection	1	3	6	10	14
Open Treatment of Femoral Shaft Fracture	2	3	5	7	10
Total	191	237	275	318	382

## Discussion

Case volume is an increasingly scrutinized metric during fellowship training, given its implications on surgical outcomes [5-15]. While case minimum requirements do not exist for ACGME accredited fellowship training, there has been discussion to further standardize training in pediatric orthopedic surgery [18,19]. Importantly, to receive Pediatric Orthopaedic Society of North America (POSNA) accreditation, programs are required to provide fellows with a minimum of 250 operative orthopedic cases per fellow. Recently trained orthopedic surgeons can expect to effectively double their pediatric case experience at the completion of ACGME accredited pediatric orthopedic fellowship training. Further research is needed to understand the clinical implications of more case volume on clinical competency and establish evidence-based thresholds for case minimum requirements during pediatric orthopedic fellowship training. Historically, ACGME defined case minimum requirements were determined by Delphi method based on historic benchmarks of reported case volumes. Future studies should incorporate trainee reported outcomes, including satisfaction, comfort with cases, and technical proficiency. Ultimately, the gold standard study would correlate trainee case volume with clinical outcomes, including clinical and radiographic metrics as well as Milestones assessments.

The ACGME requires 200 pediatric cases to be performed at the conclusion of orthopedic surgery residency [4]. In our study, contemporarily trained orthopedic surgery residents performed 282 pediatric cases at the conclusion of residency. The ACGME residency review committee for orthopedic surgery created case minimum requirements based on historical benchmarks from residents graduating during 2007-2008 to 2009-2010 academic years [4]. Case minimum requirements do not currently exist for pediatric orthopedic fellowship training but are a topic of ongoing discussion [19]. Future research should understand surgical learning curves for common procedures in pediatric orthopedic surgery (e.g., supracondylar humerus fractures). Only then can case minimum requirements for pediatric orthopedic surgery be established in an evidence-based manner.

The etiology of increasing case volumes in pediatric orthopedic surgery during residency training remains unclear. It may be that residents are simply reporting more of their existing cases, given the increasing scrutiny of case volumes in surgical training. A previous study described the wide variability in pediatric orthopedic surgery cases performed during orthopedic surgery residency from 2007 to 2013 [20]. The 90^th^ percentile of residents performed 3.1 times more pediatric orthopedic surgery cases than the 10^th^ percentile of residents. Interestingly, a separate study of pediatric orthopedic fellows found a 3.2-fold difference between the 90^th^ and 10^th^ percentiles of fellows regarding case volume [16]. In our study, there was a 2.0-fold difference between the 90^th^ and 10^th^ percentiles of pediatric orthopedic fellows, which suggests that this disparity has been decreasing with time. Perhaps increased training in fellow coding and the standardization of training accounts for this narrowing trend over time. Previously, only 39% of pediatric orthopedic fellows reported receiving formal instruction in coding during fellowship training. Furthermore, pediatric orthopedic fellows assigned CPT codes to five hypothetical cases that varied between 29% to 100%, thus highlighting potential limitations in surgical case log data.

With technological advancements, including the use of robotics and minimally invasive techniques in the spine and hip surgeries, scrutiny of operative training will increase for future pediatric orthopedic surgeons. Pediatric orthopedic surgery will continue to sub-specialize as the number of dual-fellowship trained surgeons increases in pediatric orthopedic surgery [21,22]. Continued work hour restrictions and other barriers to surgical education, like viral pandemics, will make achieving adequate surgical volume increasingly difficult [23]. Thus, periodic assessments of surgical case volumes obtained during training will be necessary to calibrate the utility of pediatric orthopedic fellowship training. Overall, graduates from pediatric orthopedic fellowship appear well-satisfied with the training investment [24].

There were several limitations to this study. First, only ACGME accredited pediatric orthopedic fellowships were included, which accounted for the majority of all programs. Second, case logs contain unary data, which do not capture the qualitative hands-on experience trainees received during the case. Different levels of autonomy are afforded based on perceived competency and attending surgeon comfort, which are not reflected in case volume data. Fellows are generally afforded higher levels of autonomy compared with residents, which may further amplify the educational impact of pediatric orthopedic fellowship training. Third, the clinical impact of more case volume during surgical training remains unknown. Future studies are needed to establish the impact of more cases performed during pediatric orthopedic fellowship training on surgical outcomes. Fourth, more granular temporal data on a weekly or monthly basis were unavailable, which precluded additional analyses. Lastly, case volume data is susceptible to coding inaccuracies and misrepresentation [25,26]. However, case logs are routinely monitored and audited by program directors and national accrediting bodies in orthopedic surgery education.

## Conclusions

In conclusion, pediatric orthopedic surgeons double their pediatric case volume after fellowship training. Prospective trainees and faculty are provided a benchmark to improve operative training in pediatric orthopedic surgery during residency and fellowship training. More research is needed to understand the impact of case volume on clinical outcomes in pediatric orthopedic surgery, including the potential establishment of case minimums in an evidence-based manner.
